# *Drosophila melanogaster* retrotransposon and inverted repeat-derived endogenous siRNAs are differentially processed in distinct cellular locations

**DOI:** 10.1186/s12864-017-3692-8

**Published:** 2017-04-17

**Authors:** Andrew W. Harrington, Michael R. McKain, Daniel Michalski, Kaylyn M. Bauer, Joshua M. Daugherty, Mindy Steiniger

**Affiliations:** 1 Department of Biology, University of Missouri, St. Louis, MO 63121 USA; 2 Donald Danforth Plant Science Center, 975 North Warson Road, St. Louis, MO 63132 USA

**Keywords:** Dicer2, mRNA 3’ end processing, Endogenous small interfering RNA biogenesis, Core cleavage complex, Symplekin, CPSF73

## Abstract

**Background:**

Endogenous small interfering (esi)RNAs repress mRNA levels and retrotransposon mobility in Drosophila somatic cells by poorly understood mechanisms. 21 nucleotide esiRNAs are primarily generated from retrotransposons and two inverted repeat (hairpin) loci in Drosophila culture cells in a Dicer2 dependent manner. Additionally, proteins involved in 3’ end processing, such as Symplekin, CPSF73 and CPSR100, have been recently implicated in the esiRNA pathway.

**Results:**

Here we present evidence of overlap between two essential RNA metabolic pathways: esiRNA biogenesis and mRNA 3' end processing. We have identified a nucleus-specific interaction between the essential esiRNA cleavage enzyme Dicer2 (Dcr2) and Symplekin, a component of the core cleavage complex (CCC) required for 3' end processing of all eukaryotic mRNAs. This interaction is mediated by the N-terminal 271 amino acids of Symplekin; CCC factors CPSF73 and CPSF100 do not contact Dcr2. While Dcr2 binds the CCC, Dcr2 knockdown does not affect mRNA 3' end formation. RNAi-depletion of CCC components Symplekin and CPSF73 causes perturbations in esiRNA abundance that correlate with fluctuations in retrotransposon and hairpin esiRNA precursor levels. We also discovered that esiRNAs generated from retrotransposons and hairpins have distinct physical characteristics including a higher predominance of 22 nucleotide hairpin-derived esiRNAs and differences in 3' and 5' base preference. Additionally, retrotransposon precursors and derived esiRNAs are highly enriched in the nucleus while hairpins and hairpin derived esiRNAs are predominantly cytoplasmic similar to canonical mRNAs. RNAi-depletion of either CPSF73 or Symplekin results in nuclear retention of both hairpin and retrotransposon precursors suggesting that polyadenylation indirectly affects cellular localization of Dcr2 substrates.

**Conclusions:**

Together, these observations support a novel mechanism in which differences in localization of esiRNA precursors impacts esiRNA biogenesis. Hairpin-derived esiRNAs are generated in the cytoplasm independent of Dcr2-Symplekin interactions, while retrotransposons are processed in the nucleus.

**Electronic supplementary material:**

The online version of this article (doi:10.1186/s12864-017-3692-8) contains supplementary material, which is available to authorized users.

## Background

In *Drosophila,* independent groups of small RNAs with overlapping function regulate gene expression using transcriptional and post-transcriptional mechanisms. PIWI-interacting RNAs (piRNAs) are found, most notably, in the germ line where they inhibit transposon (Tn) expression by inducing heterochromatin formation at complementary genomic Tn insertion sites [1–8]. Micro RNAs (miRNAs) and endogenous small interfering RNAs (esiRNAs) are expressed ubiquitously; however miRNAs frequently inhibit translation of protein coding genes [9], while esiRNAs are suggested to inhibit Tn mobility in *Drosophila* somatic cells [4–6] and potentially target mRNAs for degradation using a cytoplasmic RNAi mechanism [10, 11]. While PIWI mediated Tn repression in germ cells and translational inhibition by miRNAs have been actively investigated, the molecular details of how esiRNAs regulate their targets have not been described.

Twenty-one nucleotide (nt) esiRNAs are generated from double stranded (ds) precursor RNAs by Dicer-2 (Dcr2) and function through association with Argonaute-2 (Ago2) in *Drosophila* somatic cells [11–16]. esiRNAs produced in *Drosophila* tissues derive generally from *cis*-natural antisense transcripts (*cis*-NATs), inverted repeat containing single stranded RNAs (hairpins (hps)), and retroTns [11, 12, 14, 15]. In contrast, *Drosophila* culture cells generate esiRNAs predominantly from long terminal repeat (LTR) retrotransposons (retroTns) and hps; few *cis*-NAT derived esiRNAs are observed in S2 cell derived datasets [12, 13, 17]. Multi-copy LTR and non-LTR retroTns generate esiRNAs that map the entire length of these retroTns. In contrast, hp-derived esiRNAs arise from two loci, defined Esi1 and Esi2, within *Drosophila* annotated transcripts CR18854 and CG47744, respectively. Esi1 and Esi2 contain multiple inverted repeats, allowing formation of complex dsRNA secondary structures. These loci produce multiple esiRNAs, the most predominant termed Esi1.2 and Esi2.1. Differences between retroTn and hp-derived esiRNA biogenesis have not been previously investigated.

*Drosophila* LTR and non-LTR retroTns are transcribed in both the sense (S) and antisense (AS) directions from RNA polymerase II-like promoters [17]. S retroTn transcripts are generally polyadenylated while AS transcripts are less likely to contain a poly(A) tail [17]. Because retroTns are polyadenylated, the 3’ ends of potential esiRNA precursors are processed by the core cleavage complex (CCC) containing CPSF73, CPSF100 and Symplekin, [18–20] since this complex cleaves all eukaryotic mRNAs. Potential connections between mRNA 3’ end processing and esiRNA biogenesis are intriguing and have not been previously described.

esiRNAs regulate Tns and additional targets via multiple pathways: A canonical cytoplasmic post-transcriptional RNAi pathway in which esiRNAs hybridize to target mRNAs resulting in translational repression, and/or transcriptional regulation by induction of heterochromatin in the nucleus. mRNA targets of hp derived esiRNAs have been identified [11] and transcript levels of these targets are elevated in *Dcr2* mutant flies, [10] supporting the post-transcriptional model. Evidence is mounting that Tn derived esiRNAs also mediate heterochromatin formation in *Drosophila* nuclei [1, 5–7]. *Dcr2* catalytic mutants regulate position effect variegation [6, 7], a measure of heterochromatin formation [21, 22]. Additionally, Dcr2 promotes transcription of heat shock genes [23] and has been observed in the nuclei of *Drosophila* larvae [24]. These data are consistent with a nuclear pool of Dcr2 that could contribute to transcriptional regulation by induction of heterochromatin in addition to cytoplasmic Dcr2 acting in the RNAi pathway.

To define connections between differential retroTn and hp-derived esiRNA processing and cellular location, and to investigate the potential link between mRNA 3’ end cleavage and esiRNA biogenesis, interactions between CCC components and Dcr2 were characterized and esiRNAs in control and RNAi-depleted *Drosophila* tissue culture cells were analyzed. These experiments revealed that Dcr2 and the CCC interact, but only in the nucleus, and that the CCC indirectly regulates esiRNA biogenesis by modulating dsRNA precursor levels. Additionally, retroTn- and hp-derived esiRNAs are physically distinct and occupy different subcellular compartments. RetroTn-derived esiRNAs and their precursors are retained in the nucleus while hp-derived esiRNAs and their precursors are exported to the cytoplasm. Collectively, these data support a model in which esiRNAs regulate gene expression and retroTn mobility via diverse compartmentalized mechanisms.

## Methods

### Stable expression of Symplekin mutants, RNAi

Creation of Dmel-2 stable cell lines expressing full-length, N- and C-terminal Symplekin mutants was performed as described [20]. RNAi was performed as described [17].

### Crude nuclear and refined nuclear/cytoplasmic extracts

Crude nuclear extracts were prepared as described [18]. Refined nuclear and cytoplasmic fractions were prepared as described [18, 25] with the following modifications. 500 x 10^6^ cells were collected, washed 2X with cold PBS, re-suspended in 5X the cell pellet volume of hypotonic buffer (10 mM HEPES/KOH, pH 7.9, 1.5 mM MgCl_2_, 10 mM KCl, 0.5 mM DTT) and incubated on ice for 30 min. Cells were lysed with a 15 mL tight glass dounce tissue homogenizer 20X. Lysate was spun at 17 K x g at 4 °C for 10 min to pellet crude nuclei. Supernatant was removed, spun again to remove any residual debris, snap frozen in Liquid N_2_ and saved as the refined cytoplasmic fraction. The crude nuclear pellet was resuspended in 2 mL S1 buffer (20 mM HEPES/KOH, pH 7.9, 0.88 M sucrose, 5 mM MgCl_2_, 0.5 mM DTT) layered on top of 15 mL S2 buffer (20 mM HEPES/KOH, pH 7.9, 2 M sucrose, 5 mM MgCl_2_, 0.5 mM DTT) and spun at 18.5 K x g for 30 min at 4 °C to pellet nuclei. Buffers S1 and S2 were removed. The refined nuclear pellet was resuspended in 500 μL of cold PBS and spun for 5 min at 18.5 K x g at 4 °C to repellet the nuclei. For IPs and western blots, the refined nuclear pellet was lysed in a high salt buffer as described [18]. Both refined nuclear lysate and refined cytoplasmic lysate were dialyzed overnight in Buffer D (20 mM HEPES/KOH, pH 7.9, 20% glycerol, 100 mM KCl, 0.2 mM EDTA, 0.5 mM DTT). For RT-qPCR, RNA was extracted using 1 mL (nuclear pellet) or three volumes (cytoplasmic lysate) Trizol reagent (Ambion).

### Immunoprecipitation, western blotting, S1 nuclease assay

Immunoprecipitation of HA-tagged proteins was performed as described [20]. Immunoprecipitation of endogenous proteins was performed as described [18] using 100 μg of crude nuclear or 175 μg refined cytoplasmic or nuclear extracts. S1 nuclease protection assay was performed as described [20]. Monoclonal and polyclonal HA antibodies (Cat#s MMS-101R and PRB-101C, respectively, Covance) were used for both IP (3 μL) and WB (1:1000). Anti-CPSF73, anti-Symplekin, and anti-CPSF100 antibodies (1:1000) were described previously [18, 26]. Commercial anti-Dcr-2 (Abcam ab4732), anti-Actin (Abcam ab8227), anti-H3 (Cell Signaling 4499), and anti-MEK1/2 (Cell Signaling 8727) were used at manufacturer recommended concentrations. The anti-R2D2 antibody was a generous gift from the Siomi lab [27].

### RT-qPCR from nuclear and cytoplasmic fractions

Nuclear/cytoplasmic enrichment analysis of precursors and esiRNAs by RT-qPCR used Trizol prepped total RNA from the refined fractionation. All samples were column cleaned using the Qiagen miRNeasy Mini Kit (217004) and DNAse treated (Ambion Turbo DNAse # AM 1907) prior to RT. Equal cellular volumes were used in the RT step. RT-qPCR of precursors utilized iScript Reverse Transcription Supermix and SsoAdvanced Universal SYBR Green (Biorad #170884, #1725271, respectively). siRNA RT-qPCR was performed using Taqman Micro RNA RT Kit and Taqman Universal Master Mix (AB #4366596, #4440040, respectively.) Custom small RNA assay numbers and PCR primers are listed in Additional file 1. All qPCR experiments were performed in triplicate.

### Immunofluorescence

Immunofluorescence was performed essentially as described [28]. Nuclei were stained with DAPI. Anti-Symplekin [18] was used at 1:500 and anti-Dcr-2 [29] was used at 1:200. Secondary antibodies were used at 1:1000. Images were obtained on a Zeiss LSM 700, maintaining equal laser strength, gain and 1 AU. The images were processed with ImageJ.

### rRNA depletion, library preparation and high-throughput sequencing

rRNA depletion, library preparation and next generation sequencing RNA-seq/small RNA-seq was performed as described [17]. smRNA libraries were constructed from biological duplicates. One biological siRNA-seq and the RNA-seq were performed in technical triplicate.

### HTS analysis

siRNAs were analyzed using a newly developed pipeline called SMACR (*S*equence *M*apping, *A*nnotation, and *C*ounting for sm*R*NAs; https://github.com/mrmckain/SMACR). Raw reads were first trimmed using Trimmomatic v.0.33 [30], with parameters optimized for siRNA data: Adapter trimming using TruSeq3-SE adapters, seed mismatch of 1, palindrome clip threshold of 20, and simple clip threshold of 7; a quality sliding window of 3 basepairs (bp) with a minimal average score of 20; and a minimum length of 19. Trimmed reads were then filtered to remove any longer than 30 bp. Relative abundances were then calculated for all unique trimmed reads. Unique is a read that is different from all others. The unique reads were then mapped to the *Drosophila melanogaster* genome (Dmel v.6.01; [31]) using bowtie v.1.1.1 [32] allowing for either 0 or 1 mismatches. The mapping and read abundance information were then merged, estimating reads per million (rpm) for each mapped unique sequence. SMACR can simultaneously read in multiple experimental datasets, including replicates, and maintains each dataset as uniquely identified to the particular experiment and replicate. Annotation coordinates from Dmel v.6.01 for miRNAs, noncoding RNAs, transposons, and two hairpin structures were used to link mapped siRNAs to annotation features. If a siRNA was found to map to more than one feature type, it was disregarded. Abundance (normalized read counts) of siRNAs mapping to a particular feature were totaled and percentages of siRNAs mapping to each feature were calculated for each replicate. 5’ and 3’ nucleotide abundance, siRNA abundance, and relative phasing to the core siRNA for a given mapping site were then analyzed in the final set of siRNAs. Averages include technical triplicates and the biological replicate and standard deviations reflect the standard error of the mean for all four samples. RNA-seq reads from Symplekin and CPSF73 depleted samples were strand specifically mapped using the RNA-seq Unified Mapper (RUM) [33] and visualized with the University of California Santa Cruz (UCSC) genome browser (http://genome.ucsc.edu, Dm6 assembly, August 2014) [31, 34]. Further analyses were performed as in [17].

### Poly(A)+/- analysis

Poly(A)+/- analysis and calculations were performed as in [17].

## Results

### mRNA 3’ end processing factor Symplekin interacts with Dcr2

**Fig. 1:**
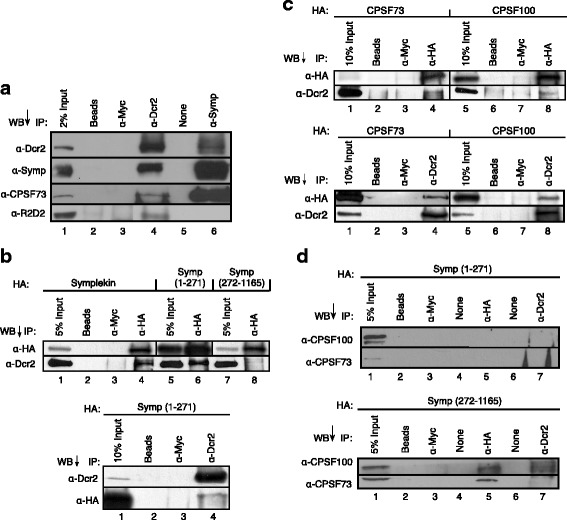
Dcr2 interacts with the N-terminal region of Symplekin **a** Dcr2 co-immunoprecipitates (co-IP) with the core cleavage complex (CCC) and R2D2 in *Drosophila* culture cell crude nuclear extract. Antibodies used for immunoprecipitation (IP) are shown above. Antibodies used for western blot (WB) are listed to the left. ‘Beads’ and ‘α-Myc’ are negative controls. No sample was loaded in lanes labeled ‘None.’ **b** Dcr2 binds Symplekin amino acids 1-271 and not amino acids 272-1165. Exogenously expressed HA-tagged Symplekin deletions are defined above the blots. Other labels are as in **a**. WB of full length Symplekin and Symplekin mutant IPs are the top figure while co-IP of Symp(1-271) with Dcr2 is shown in the bottom WB. **c** Dcr2 binds exogenously expressed CCC components CPSF73 and CPSF100. HA-tagged, full-length CPSF73 and CPSF100 were IPed from *Drosophila* culture cells stably expressing these proteins. Dcr2 co-IPs with both CPSF73 and CPSF100 was identified by western blot (top). Other labels are as in **a**. Dcr2 was IPed from these cells. Co-IP of HA-CPSF73 and HA-CPSF100 was identified by western blot (bottom). Controls are as in **a. d** CCC components CPSF100 and CPSF73 do not interact with Dcr2 in the absence of full length Symplekin. WB of Symp(1-271) IP (top) and WB of Symp (272-1165) IP (bottom) from Symplekin RNAi-depleted samples are shown. WB are labeled as in **a**

To identify potential novel CCC binding partners, we immunoprecipitated endogenous Symplekin from crude *Drosophila* culture cell nuclear extracts and identified co-immunoprecipitating proteins by mass spectrometry (Additional file 2). The most abundant Symplekin interacting proteins in this assay were known CCC components CPSF73 and CPSF100 and additional mRNA 3’ end processing proteins CPSF160, WDR33 (CG1109), [35, 36] CPSF6 (CG7185), and CstF77 [37]. Surprisingly, Dcr2 and Hsc70, proteins known to act in siRNA biogenesis [16, 38] also interacted with Symplekin (Additional file 2). To confirm this interaction, we performed the reverse immunoprecipitation. Dcr2 co-immunoprecipitated Symplekin and additional CCC factor components, CPSF73 and CPSF100, and R2D2, a known Dcr2 binding partner [39] (Fig. 1a). Additionally, endogenous Dcr2 co-immunoprecipitated with HA-tagged Symplekin (Fig. 1b), CPSF73 and CPSF100 (Fig. 1c) stably expressed in Dmel-2 cells. When endogenous Dcr2 was immunoprecipitated from these cells, HA-CPSF73 and HA-CPSF100 co-immunoprecipitated indicating that Dcr2 interacts with the CCC (Fig. 1c).

To determine which region of Symplekin interacts with Dcr2, we immunoprecipitated stably expressed HA-tagged Symplekin deletions from *Drosophila* culture cell lysates. The N-terminal region of Symplekin (amino acids 1-271) clearly interacts with endogenous Dcr2 while the C-terminal region (amino acids 272-1165) does not (Fig. 1b). Reciprocal immunoprecipitation of endogenous Dcr2 reveals co-immunoprecipitation of HA-tagged Symp (1-271) (Fig. 1b).

To further investigate Dcr2-CCC interactions, we used a system in which N- and C-terminal Symplekin mutants are expressed in endogenous Symplekin RNAi-depleted cells. CCC formation is mediated by the C-termini of Symplekin, CPSF73 and CPSF100 [20]. Therefore, exogenous HA-Symp (1-271) does not precipitate endogenous CPSF73 or CPSF100 when full-length endogenous Symplekin is knocked down (Fig. 1d). Unlike the interactions observed with full-length Symplekin, Dcr2 does not immunoprecipitate CPSF73 and CPSF100 under conditions when only HA-Symp (1-271) is present (Fig. 1d). Additionally, while CCCs are formed in cells expressing HA-Symp (272-1165), very little CPSF73 and CPSF100 interact with Dcr2 in these cells (Fig. 1d). These data suggest that the Symplekin N-terminal region interacts with Dcr2 while CPSF73 and CPSF100 are present in this complex via interaction with the C-terminal region of Symplekin [20].

### The Dcr2-CCC complex is functionally distinct from the CCC

**Fig. 2:**
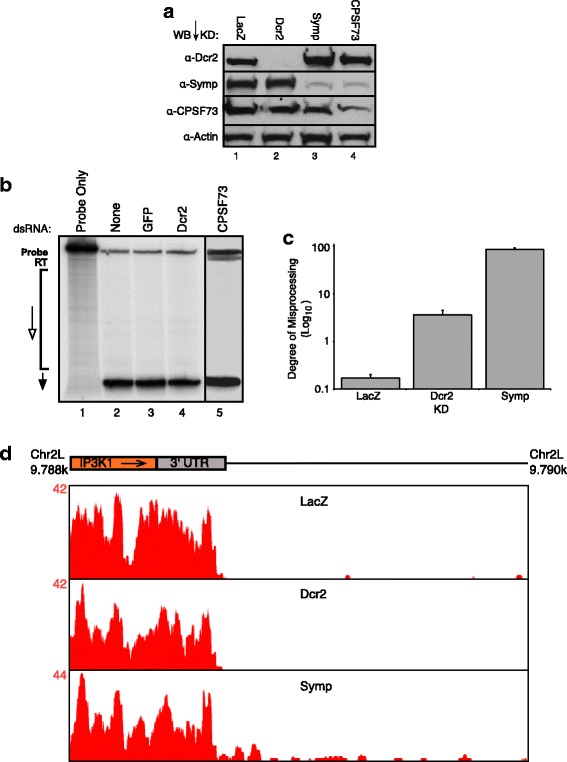
Dcr2 is not involved in mRNA 3’ end processing **a** Dcr2 depletion does not affect CCC component protein levels. RNAi-depleted proteins are listed above the blot. Antibodies used for WB are listed to the left. **b** Dcr2 RNAi-depletion does not cause mRNA 3’ end misprocessing. An S1 nuclease assay was used to map histone (H)2A 3’ ends (left). Knockdowns are shown at the top. Potential mRNA 3’ end products are shown to the left: RT is the read-through misprocessed product, the open arrow marks the region of other misprocessed products, and the black arrow defines the properly processed product. CPSF73 is shown as a positive control. **c** RT-qPCR using primers that amplify misprocessed *sop* mRNAs (right) reveals very little misprocessed *sop* in Dcr2 knockdown samples. Knockdowns are shown on the x-axis. Degree of Misprocessing = Log_10_ (2^ΔΔCt(ORF-MP)). Error bars represent one standard deviation. **d** Dcr2 RNAi-depletion does not cause 3’ end misprocessing and read-through transcription. RNA-seq reads mapping to and downstream of the IP3K1 gene are shown in red. Maximal read counts for IP3K1 are shown on the y-axis

CPSF73, CPSF100 and Symplekin tightly interact in the absence of RNA to form the CCC [18]. Dcr2 and Symplekin also interact in the absence of RNA (Andrew Harrington, data not shown). When one member of the CCC is depleted, levels of the other factors are dramatically reduced [18]. To determine if Dcr2 is a bona fide CCC component, we investigated Symplekin and CPSF73 levels in a Dcr2 knockdown. When Dcr2 is depleted, Symplekin and CPSF73 levels are unchanged (Fig. 2a). Dcr2 levels remain constant when CPSF73 or Symplekin are knocked down (Fig. 2a).

Because CCC depletion causes 3’ end misprocessing and Dcr2 interacts with the CCC, we wanted to determine the effects of Dcr2 depletion on mRNA 3’ end processing. First, we mapped the 3’ ends of endogenous Histone 2A (H2A) mRNAs in a Dcr2 depleted sample using an S1 nuclease protection assay. No differences in mRNA 3’ end processing were observed between Dcr2 knockdown and negative control samples (Fig. 2b). Dcr2 depletion does not cause misprocessing of histone mRNA 3’ ends as is observed when CCC components are knocked down (Fig. 2b) [18, 20]. Additionally, an RT-qPCR assay [40] was used to assess the effects of Dcr2 knockdown on mRNA 3’ end misprocessing of polyadenylated genes. Very little misprocessing of a canonical polyadenylated mRNA (*sop*) was observed in a Dcr2 depleted sample as compared to the positive Symplekin knockdown control; compare ~4-fold in the Dcr2 knockdown to ~85-fold in the Symplekin RNAi-depleted sample (Fig. 2c). Finally, visual inspection of mapped RNA-seq reads from control (LacZ), Dcr2 and Symplekin knockdown samples reveals mRNA 3’ end misprocessing is only present in the Symplekin RNAi-depleted sample; Dcr2 and LacZ knockdown samples show no or few RNA-seq reads mapping downstream of the IP3K1 gene (Fig 2d). These data support a model in which Dcr2 is not required for mRNA 3’ end processing and that the Dcr2-CCC complex is functionally distinct from the CCC as mRNA 3’ end processing is unaffected in the absence of Dcr2.

### Dcr2 interacts with the CCC in the nucleus

**Fig. 3:**
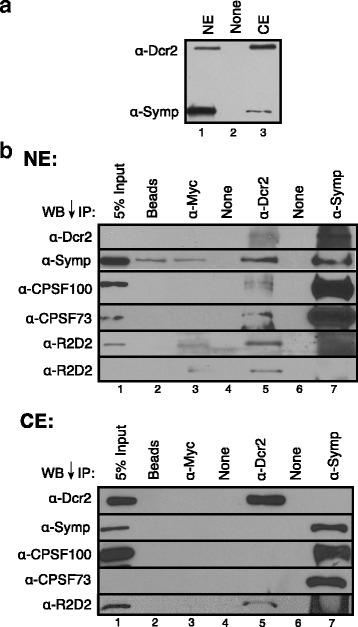
Dcr2 only interacts with the CCC in the nucleus. **a** Dcr2 is present in the nucleus. WB of refined nuclear (NE) and cytoplasmic extracts (CE) reveals a nuclear pool of Dcr2 (top). **b** Endogenous Dcr2 co-IPs the CCC and R2D2 from refined NEs (top). No interaction between Dcr2 and the CCC is observed in CE (bottom). Antibodies used for IP are shown above. Antibodies used for WB are listed left. ‘Beads’ and ‘α-Myc’ are negative controls. A lighter exposure of the R2D2 WB is shown at the bottom of the top NE group

To investigate subcellular localization of the Dcr2-CCC complex, Dmel-2 cells were first effectively separated into cytoplasmic and nuclear fractions using a refined fractionation technique (Methods, Additional file 3). Western blots reveal pools of Dcr2 and Symplekin in both the nucleus and the cytoplasm (Fig. 3a). While Dcr2 is primarily cytoplasmic and Symplekin is generally nuclear in accordance with their roles in RNAi and mRNA 3’ end processing, respectively, an appreciable amount of each protein is found in the complementary subcellular compartment (Fig. 3a). Additionally, immunofluorescence with antibodies to the endogenous proteins confirms the presence of both Symplekin and Dcr2 in the nucleus (Additional file 4). This assay also shows Symplekin and Dcr2 in the cytoplasm, consistent with their roles in cytoplasmic polyadenylation and RNAi, respectively [27, 41, 42].

Immunoprecipitations of endogenous Dcr2 from refined Dmel-2 nuclear and cytoplasmic fractions show that nuclear Dcr2 co-immunoprecipitates the CCC and R2D2 (Fig. 3b), while cytoplasmic Dcr2 only interacts with R2D2 (Fig. 3b); no interaction between cytoplasmic Dcr2 and the CCC is observed. Nuclear Symplekin co-immunoprecipitates Dcr2 and other CCC components, CPSF73 and CPSF100, but not R2D2 (Fig. 3b). Additionally, cytoplasmic Symplekin does not interact with Dcr2 (Fig. 3b). Together these data support a model in which Dcr2 forms distinct nuclear and cytoplasmic complexes.

### The CCC indirectly regulates esiRNA abundance

To investigate the role of the CCC in esiRNA biogenesis, CPSF73, Symplekin and Dcr2 were first independently RNAi-depleted from *Drosophila* culture cells. Untreated Dmel-2 cells (blank) or those treated with double stranded RNA to a non-*Drosophila* gene (GFP or LacZ) acted as controls. RNA was then isolated and separated into large (>200 nts) and small (<200 nts) fractions, rRNA depleted and sequenced (Additional file 5). RNA-seq reads were mapped to the *Drosophila* genome and transcriptome using RUM, [33] while small RNA (smRNA)-seq reads were mapped and analyzed using a novel pipeline termed *S*equence *M*apping, *A*nnotation, and *C*ounting for sm*R*NAs or SMACR (Methods and Additional file 5). Only siRNAs and miRNAs were further analyzed. Interestingly, ~40% of RNA-seq reads mapped to multiple locations in the genome (non-unique), although the samples were depleted of rRNAs (Additional file 6). Also, the percentage of non-unique reads changes significantly with knockdown of Dcr2 and Symplekin (Additional file 6). These data support previous claims that Dmel-2 culture cells have undergone Tn expansion [43–46] and indicate that increased numbers of Tns may contribute to higher overall expression of repetitive sequences and abundance of Dcr2 dependent siRNAs. Tn expansion makes *Drosophila* culture cells an excellent system for studying esiRNAs biogenesis.

**Fig. 4:**
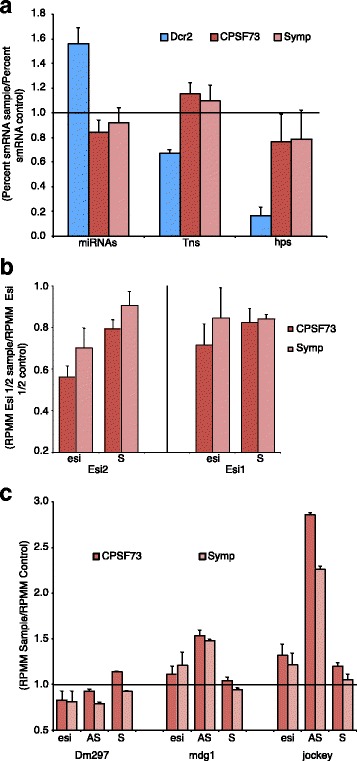
CCC depletion differentially affects esiRNA biogenesis from retroTns and inverted repeat loci. **a** Ratios of percent miRNAs, transpsoson (Tn)-derived and hairpin (hp)-derived siRNAs from Symplekin (pink), CPSF73 (red), and Dcr2 (blue) knockdown samples are shown. Percentages are the total miRNA, Tn or hp normalized read count (reads per million mapped (RPMM)) divided by the total normalized read count (summed normalized miRNA, pre-miRNA, Tn, non-coding RNA, and hp RPMs). The percents of each smRNA group were normalized to the LacZ control. Error bars represent one standard deviation. **b** CPSF73 and Symplekin RNAi-depleted samples are represented as in **a**. RPMMs of esiRNAs mapping to hp loci Esi1 and Esi2 in Symp and CPSF73 depleted samples were normalized to corresponding esiRNAs in LacZ samples (left). RPMMs of RNA-seq reads mapping to sense (S) precursors of Esi1 and Esi2 RNA in these knockdowns were also normalized to corresponding RNA-seq reads in LacZ samples (right). Error bars are as in **a. c** RPMMs of esiRNAs mapping to Dm297, mdg1 and jockey retroTns in Symp and CPSF73 depleted samples were normalized to corresponding esiRNAs in LacZ samples (left). RPMMs of RNA-seq reads mapping to sense (S) and antisense (AS) precursors of Dm297, mdg1, jockey transcripts in these knockdowns were also normalized to corresponding RNA-seq reads in LacZ samples (right). Error bars are as in **a**

We first assessed how depletion of Dcr2 and CCC components CPSF73 and Symplekin affect siRNA and miRNA dynamics in *Drosophila* culture cells. First, reads per million mapped (RPMM) pre-miRNAs, non-coding (nc)RNAs, miRNAs, Tn-mapping esiRNAs, and hairpin structure-mapping (Esi1/2 (hps)) esiRNAs, were added to give the total smRNA pool for each sample. The percentages of miRNAs, Tn-mapping and hp-mapping esiRNAs were then calculated for each sample. Normalizing to the total number of smRNAs is important as Symplekin, CPSF73 and Dcr2 knockdown samples have decreasing amounts of total smRNAs (data not shown). To evaluate CCC and Dcr2 based differences in miRNA, Tn-mapping, and hp-mapping esiRNA levels, the percentage of smRNAs in each group was divided by the percentage of each smRNA group in the LacZ control (Fig. 4a). When Dcr2 is RNAi-depleted, the percentage of esiRNAs mapping to Tns and hps decreases significantly while the portion of miRNAs in the pool increases (Fig. 4a). Biogenesis of hp esiRNAs is more dependent on Dcr2 than esiRNAs processed from Tn precursors, as Dcr2 depletion reduces hp esiRNAs ~7.3 fold compared to the control, while Tn esiRNAs are only reduced ~1.3 fold (Fig. 4a). Surprisingly, depletion of CCC components CPSF73 and Symplekin has differential effects on Tn and hp derived esiRNAs; the proportion of Tn derived esiRNAs generally increases while the number of esiRNAs generated from hps trends downward (Fig. 4a). Knockdown of Symplekin and CPSF73 may slightly reduce the number of miRNAs in these samples (Fig. 4a). Importantly, RNAi-depletion of CPSF73 and Symplekin show similar trends while the Dcr2 knockdown displays a different molecular phenotype. Together these data support a model in which esiRNAs are differentially processed from Tn and hp precursor molecules in *Drosophila* culture cells.

To investigate potential explanations for the observed differences between Tn and hp derived esiRNA levels and differential effects of Dcr2 and CCC factor depletion on esiRNAs biogenesis, we first examined esiRNA and precursor levels for both hp loci (Esi 1/2) and individual retroTns: Dm297, mdg1 and jockey, as compared to the LacZ control (Fig. 4b and c). Normalized Esi 1/2 precursor and esiRNA levels in each knockdown sample were divided by the number of Esi 1/2 precursor and esiRNAs in the LacZ control. Esi 1/2 precursor levels decrease when CPSF73 and Symplekin are knocked down and we observe a corresponding decrease of esiRNAs in these samples (Fig. 4b). The number of esiRNAs mapping to mdg1 and jockey increase in response to CCC depletion while esiRNAs generated from Dm297 decrease in these samples (Fig. 4c). RetroTn esiRNA precursors consist of hybridized S and AS retroTn transcripts [17]. We previously reported that both S and AS retroTn transcript levels are elevated in Dcr2 depleted cells [17]. Knockdown of CCC components Symplekin or CPSF73 results in little to no change in sense Dm297, mdg1 or jockey transcript abundance, while the corresponding AS transcript levels are altered significantly (Fig. 4c). Interestingly, retroTn esiRNA levels correlate with perturbations in AS transcript levels in these samples (Fig. 4c). Previously, we hypothesized that the total amount of retroTn Dcr2 substrate in Dmel-2 cells is determined by AS transcript levels as these tend to be limiting [17]. Therefore, changes in retroTn derived esiRNAs levels partially correspond to alterations in retroTn Dcr2 substrate levels. But, because changes in Esi 1/2 and retroTn esiRNA levels do not mirror alterations in substrate abundance (Fig. 4b and c), additional direct affects of CCC-Dcr2 interaction are plausible and require further investigation.

**Fig. 5:**
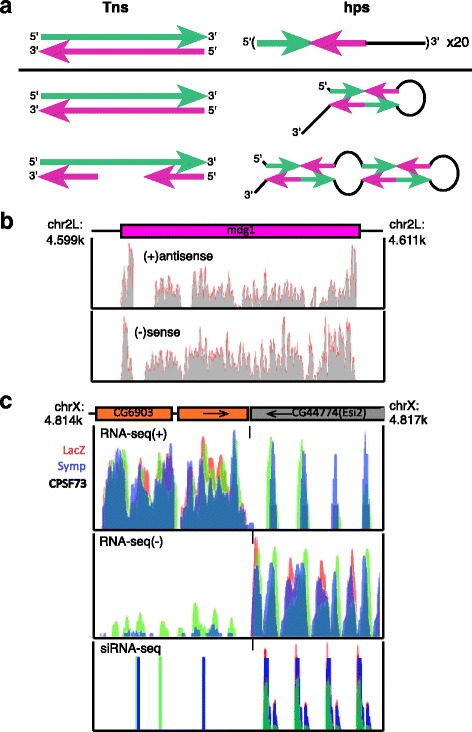
CCC factor RNAi-depletion may alter hairpin secondary structure **a** Potential secondary structures for Tns (left) and hps (right). Complementary regions are shown in green and magenta. **b** Depletion of CCC components does not cause 3’ end misprocessing of retroTn transcripts. S and AS RNA-seq reads from LacZ (control), Symplekin, and CPSF73 RNAi-depleted samples were visualized using the UCSC genome browser and are overlayed. Schematic of a genomic region containing a mdg1 element is shown above the bedgraph. No unique reads are observed flanking the retroTn. **c** CG6903 read-through transcripts could hybridize to Esi2 RNAs in CCC factor knockdowns. Symplekin (blue), CPSF73 (green) depleted samples and LacZ (red) control RNA mapping to this region are shown. RNA-seq data is displayed in the top two panels, siRNA-seq data is displayed in the bottom panel

We hypothesize that retroTn-derived dsRNAs in wild type Dmel-2 cells have a simple secondary structure, generally having blunt-ends with complementary S and AS strands as transcription is initiated from multiple internal locations (Fig. 5a). Theoretically, hairpins, having multiple inverted repeats can hybridize to form an infinite number of complex secondary structures (Fig. 5a). To determine if retroTn and hp esiRNA precursor structures are altered by depletion of CCC factors, we investigated 3’ end misprocessing of retroTn mdg1 and hp Esi2. Bedgraphs representing RNA-seq S and AS reads mapping to mdg1{}305 and surrounding sequences in CPSF73, Symplekin and LacZ depleted samples show no reads mapping beyond the 3’ ends of mdg1{}305, indicating that extended retroTn 3’ UTR phenotypes are not observed when CCC components are RNAi-depleted (Fig. 5b). This result is inconsistent with 3’ UTR molecular phenotypes observed for mRNAs (Fig. 2c). However, RNA-seq reads mapping downstream of Esi2-containing mRNA CG44774 and neighboring gene CG6903 are readily detectable in Symplekin and CPSF73 knockdowns (Fig. 5c), consistent with other mRNAs in CCC RNAi-depleted samples (Fig. 2c). The presence of read-through CG6903 mRNAs complementary to Esi2 could potentially alter Esi2 structure and give rise to additional dsRNA Dcr2 substrates. Together, these data indicate that CCC knockdown does not change retroTn Dcr2 substrate structure, while Symplekin and CPSF73 RNAi-depletion may indirectly lead to additional Esi2 dsRNAs formed with AS sequences. Inefficient cleavage of Esi2 dsRNAs composed of both S and AS RNAs could provide one explanation for the lower Esi2 esiRNA levels observed in CPSF73 and Symplekin knockdowns (Fig. 4b, Fig. 5c).

### Hp and Tn-derived esiRNAs are physically distinct

Initial characterization of siRNAs and miRNAs in Dcr2 RNAi-depleted, CCC knockdown, and control samples included using SMACR to filter smRNAs by length, 3’, and 5’ base. Generally, there is little variation between depleted samples and few statistically significant differences between Dcr2, CPSF73 and Symplekin RNAi-depleted samples, and controls (Additional file 7). One notable exception is that Dcr2 depletion reduces the percentage of 21 nt Tn and hairpin-derived esiRNAs, while increasing esiRNAs of other sizes (Additional file 7). Knockdown of Dcr2 does not affect miRNA length distributions (Additional file 7), indicating that this molecular phenotype is specific to Dcr2 substrates. This trend is not observed for 3’ and 5’ base preference in Dcr2 knockdown samples (Additional file 7). CCC factor knockdowns have negligible affects on esiRNA size and end nucleotide preference supporting a model that CPSF73 and Symplekin do not directly affect Dcr2 catalytic activity (Additional file 7). Very few statistically significant differences in smRNA length, 3’, or 5’ base are observed between untreated cells and those treated with non-specific dsRNA (LacZ) suggesting that inducing the RNAi response does not affect smRNA size or end nucleotide preference.

**Fig. 6:**
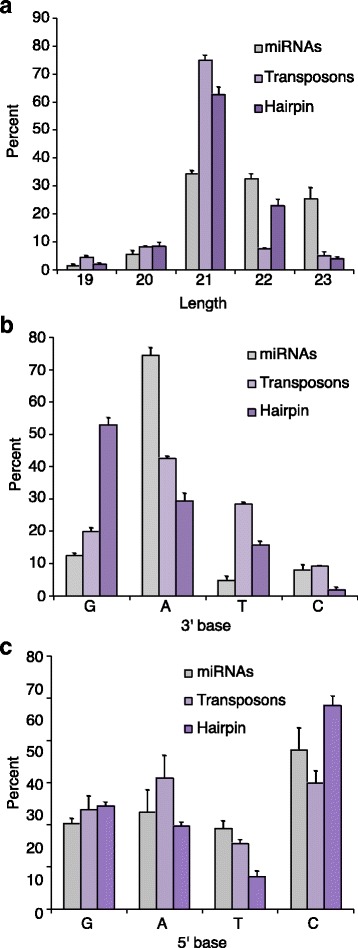
RetroTn- and hp-derived esiRNAs have different physical characteristics. **a** Percentage of miRNAs (gray), Transposons (light purple), and hairpin (purple) mapping esiRNAs in the LacZ control sample that are 19-23 nts. Error bars represent one standard deviation. **b** Percentage of miRNAs, Transposons, and hairpin mapping esiRNAs in the LacZ control sample that have a 3’ G, A, T, or C. Colors and error bars are as in **a. c** Percentage of miRNAs, Transposons, and hairpin mapping esiRNAs in the LacZ control sample that have a 5’ G, A, T, or C. Colors and error bars are as in **a**

Examination of length differences between esiRNAs and miRNAs in control samples reveals that miRNAs are almost equally distributed among 21, 22 and 23 nt lengths, while 21 nt is the dominant length of Tn and hp-derived esiRNAs (Fig. 6a). Unexpectedly, variations in length distributions were also observed between Tn and hp-derived esiRNAs. Approximately 75% of esiRNAs generated from Tns are 21 nt with 19, 20, 22 and 23-mers almost evenly comprising the remaining 25% (Fig. 6a). In contrast, ~62% of hp-derived esiRNAs are 21 nt and ~23% are 22 nt; the proportion of 22-mers in the hp generated esiRNA pool is significantly greater than for Tn-derived esiRNAs (Fig. 6a).

Dramatic differences between 3’ base preference are also observed for miRNAs and esiRNAs. The 3’ nucleotide is A for ~75% of miRNAs while C, G and T occur much less frequently at this position (Fig. 6b). Tn generated esiRNAs also predominantly end in an A, but the 3’ nucleotide is more frequently C, G and T than for miRNAs; (Fig. 6b). Interestingly, the 3’ base of hp-derived esiRNAs is G more than 50% of the time, A is observed for ~30% of these esiRNAs, and T and C are present less often at the 3’ end (Fig. 6b). Therefore, while the Tn- and hp-derived esiRNA 3’ nt is generally more diverse than for miRNAs, significant differences are observed between esiRNAs. 5’ nucleotide distributions are more similar for all three smRNA classes. C is the most abundant 5’ nucleotide for miRNAs and esiRNAs, while G and A are also frequently observed (between 25 and 40%, Fig. 6c). T is the least populous 5’ nucleotide for miRNAs and esiRNAs (Fig. 6c). Collectively, these data indicate that esiRNAs processed from Tns and hps have diverse physical characteristics and support a model in which these two precursors are differentially processed in Dmel-2 cells.

### RetroTn precursors and esiRNAs are retained in the nucleus

**Fig. 7:**
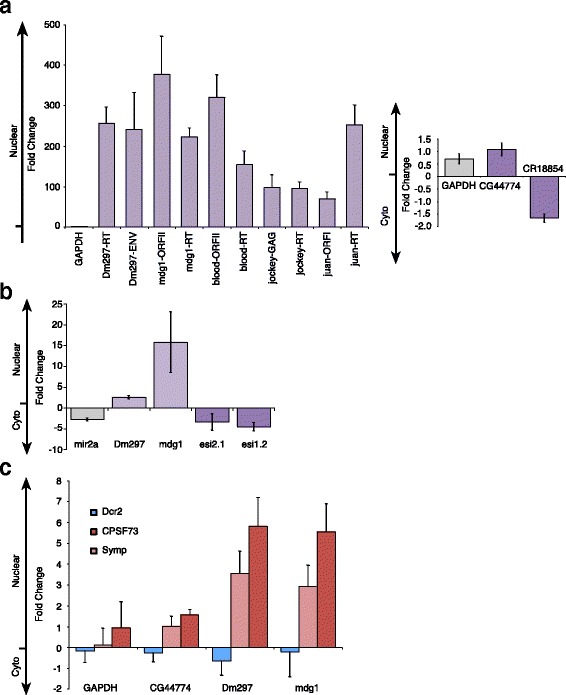
RetroTns dsRNA precursors are nuclear retained. RT-qPCR of retroTn and Esi1/2 mRNAs isolated from refined nuclear and cytoplasmic fractions reveals nuclear retention of retroTn esiRNAs precursors. (**a**, *left*) RetroTn RT-qPCR targets are shown on the x-axis. Fold change is the average of three experiments and is calculated as 2^(Ct(Nuclear)-Ct(Cytoplasm))^. (**a**, *Right*) CG44774 is the Esi1 precursor. CR18854 is the Esi2 precursor mRNA. GAPDH is the control transcript. Error bars represent one standard deviation. **b** Taqman qPCR of retroTn and Esi1/2 derived esiRNAs isolated from refined nuclear and cytoplasmic fractions shows nuclear retention of retroTn derived esiRNAs. Labels, calculations, and error bars are as in **a. c** RT-qPCR of esiRNA precursors in Symplekin, CPSF73 or Dcr2 knockdowns shows nuclear retention of both hp and retroTn dsRNA precursors. Labels, calculations, and error bars are as in **a**. RNAi-depletion is defined as in Fig. 4

To investigate potential differences in subcellular localization of retroTn and hp dsRNAs, *Drosophila* culture cells were separated into refined nuclear and cytoplasmic fractions (Additional file 3), total RNA was isolated and RT-qPCR was performed on multiple ORFs of Dm297, mdg1, blood, jockey and juan retroTn RNAs, and Esi1 and Esi2 containing substrate transcripts CG47744 and CR18854, respectively. A control canonical mRNA, GAPDH, is slightly enriched in the nucleus (Fig. 7a). Surprisingly, the retroTn transcripts are overwhelmingly enriched in the nucleus as compared to the GAPDH control showing between ~50 and ~400 fold enhancement depending on which retroTn ORF was targeted by RT-qPCR (Fig. 7a). These samples were normalized to no RT controls to ensure that contaminating genomic DNA was not responsible for the observed nuclear enhancement of retroTn RNAs. CG47744 and CR18854 are not dramatically enriched in either the cytoplasm or nucleus, resembling the GAPDH mRNA control (Fig. 7a).

To assess the cellular localization of retroTn, Esi1 and Esi2-derived esiRNAs, we measured levels of the most abundant esiRNAs from these precursors with custom Taqman assays. A cytoplasmic miRNA control (mir2A) is ~2.5 fold enriched in the cytoplasm (Fig. 7b). Strikingly, both Dm297 and mdg1-derived esiRNAs are enriched in the nucleus while Esi2 and Esi1-derived esiRNAs (Esi2.1 and Esi1.2, respectively) localize to the cytoplasm (Fig. 7b). These data correlate with nuclear enrichment of retroTns and nuclear export of hps (Fig. 7a). Together these data support a model in which retroTn dsRNA precursors are retained and processed in the nucleus while single stranded Esi1 and Esi2 precursors are exported to the cytoplasm for Dcr2-dependent generation of esiRNAs.

To investigate the role of CCC components Symplekin and CPSF73 in cellular localization of Dcr2 substrates, a subset of retroTn and hp transcript levels was assessed by RT-qPCR in refined nuclear and cytoplasmic fractions from CCC factor RNAi-depleted samples. Dcr2 knockdown has marginal effects on cellular localization of CG44774, Dm297, and mdg1 RNAs as compared to the GAPDH control. In contrast, Symplekin and CPSF73 RNA-depletions cause slight nuclear retention of hp CG44774 and further nuclear enrichment of retroTn transcripts as compared to GAPDH (Fig. 7c). While Both the CPSF73 and Symplekin knockdowns show similar trends, the effects observed when CPSF73 is RNAi-depleted are more dramatic than when Symplekin is RNAi-depleted. As both CG44774 and CR18854, and retroTn sense transcripts are polyadenylated (Additional file 8 and [17], respectively), we hypothesize that mRNA 3’ end processing defects in the CCC knockdown samples affect cellular localization of these Dcr2 substrates.

## Discussion

Since the discovery of endogenous small interfering (esiRNAs) in *Drosophila*, very little progress in understanding their biogenesis and molecular mechanisms of action has been made. Here we provide evidence that components of two major RNA processing pathways, 3’ end processing and esiRNA biogenesis, interact in *Drosophila* somatic cells, a connection not previously reported. Importantly, we also show that esiRNAs processed from retroTns have different physical characteristics than those generated from hairpins. Double-stranded retroTn RNAs are retained in the nucleus while Esi1/2 hps are exported to the cytoplasm. Together these data support a novel model in which retroTns and hps, both double stranded RNAs cleaved by Dcr2, are differentially processed in *Drosophila* somatic cells. This is the first evidence that precursor secondary structures potentially contribute to Dcr2 activity in vivo.

**Fig. 8:**
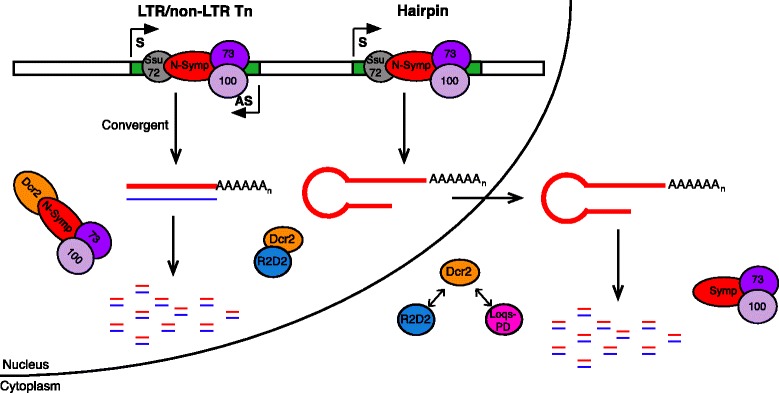
EsiRNAs are differnentially processed in *D. melanogaster* cells. Data support a model in which double stranded retroTn transcripts are retained and processed to esiRNAs in the nucleus while RNAs containing inverted repeats are exported and processed in the cytoplasm. Dcr2 interacts with the N-terminal 271 amino acids of Symplekin in the nucleus, but not in the cytoplasm

mRNA 3’ end processing performed by the CCC is co-transcriptional and therefore occurs in the nucleus [47, 48]. The RNA pol II CTD phosphatase Ssu72 interacts with the N-terminal region of Symplekin to direct processing of mRNAs with a 3’ poly(A) tail [49] and with the stem loop binding protein for replication dependent histone mRNAs (Dan Michalski, data not shown). Here, we show that this N-terminal region of Symplekin can also interact with esiRNA processing factor Dcr2 (Fig. 1) in the nuclear compartment (Fig. 3), although, Dcr2 is not required for proper mRNA 3’ end formation (Fig. 2). The Symplekin C-terminal region binds CPSF73 and CPSF100 to form the CCC [20], therefore leaving the N-terminal region free to bridge the CCC and other cellular factors. While previous work shows that regulation of Tns by piRNAs in the *Drosophila* germline is a nuclear process [50–54] and researchers have documented a nuclear pool of Dcr2 that associates with heat shock loci and transcription machinery in *Drosophila* [23], potential nuclear functions of Dcr2 in *Drosophila* somatic cells have not been extensively investigated [6]. Our data support a model in which the N-terminal region of Symplekin mediates Dcr2-CCC complex formation, but only when the CCC is not actively engaged in co-transcriptional mRNA 3’ end processing (Fig. 8).

To understand the functional implications of CCC-Dcr2 interactions, esiRNA and precursor levels were measured in Symplekin and CPSF73 RNAi-depleted samples. Globally, we observe increased levels of Tn-derived esiRNAs and decreased hp-derived esiRNAs in CCC factor knockdowns (Fig. 4). Examination of specific retroTns and Esi1/2 precursors reveals that changes in esiRNA levels correlate to shifts in precursor abundance in Symplekin and CPSF73 knockdowns (Fig. 4). As we hypothesize that dsRNA retroTn precursor levels are determined AS transcript abundance [17], more AS transcript would lead to an increase in Dcr2 substrates (and more retroTn-derived esiRNAs), while decreased AS transcript would result in less Dcr2 substrate. Esi1/2 precursors consist of only one S mRNA. Therefore, hp Dcr2 substrate concentration is determined by only CG44774 and CR18854 levels. Once again, the observed lower Esi1/2 esiRNA levels in CCC factor depleted cells correlate with decreased CG44774 and CR18854 transcripts in these samples (Fig. 4). These data support a model in which esiRNA levels are partially influenced by Dcr2 substrate concentration. Substrate levels are affected by CCC factor RNAi-depletion indicating that Symplekin and CPSF73 indirectly determine esiRNA abundance. Because Symplekin and CPSF73 RNAi-depleted samples follow the same trends, we concluded that the CCC is involved in this process.

While hp and retroTn esiRNA levels correlate to S and AS transcript abundance, the number of esiRNAs is always less than the Dcr2 substrate concentration in Symplekin and CPSF73 knockdowns indicating that additional mechanisms must be modulating esiRNA levels in these samples. Although, Dcr2 cleavage site selectivity is unaffected in CCC factor RNAi-depleted samples (Additional file 7), Dcr2 activity could be altered by interaction with the CCC. An additional hypothesis for the observed molecular phenotypes is inefficient nuclear export of retroTn and hp RNAs in Symplekin and CPSF73 RNAi-depleted samples. CCC component knockdowns cause global mRNA 3’ end processing defects (Fig. 2) and how this misprocessing affects cellular localization of retroTn dsRNAs is unknown. However, previous work shows that less polyadenylated RNAs are not effectively exported from the nucleus [55]. Additionally, 3’ end misprocessing of RNAs generated from the Esi2 locus (Fig. 5) might lead to changes in secondary structure that unpredictably affect nuclear export. Inefficient nuclear export of hp RNAs with modified 3’ ends might not change total precursor levels, but could result in less Esi2-derived esiRNAs since cytoplasmic hp precursor levels would be reduced. When Symplekin and CPSF73 are RNAi-depleted, both hp and retroTn dsRNAs are enriched in the nucleus (Fig. 7) supporting the hypothesis that non-polyadenylated RNAs are retained in the nucleus. Taken together, these data support a model in which the CCC indirectly affects the abundance of retroTn- and hp-derived esiRNAs by modulating cellular localization and concentration of Dcr2 substrates (Fig. 8).

Bioinformatic analyses of retroTn- and hp-derived esiRNAs reveals physical distinctions between these groups (Fig. 6). Additionally, retroTn precursors and their corrosponding esiRNAs are highly enriched in the nucleus while hp dsRNAs and their corresponding esiRNAs are cytoplasmic similar to mRNAs (Fig. 7). We hypothesize that these observed disparities are directly related to distinct differences in secondary structures (Fig. 5) and compartmentalization of esiRNA biogenesis factors required to process each structure. dsRNAs derived from S and AS transcription of retroTns [17] generally result in fully complementary, blunt-ended dsRNAs as many AS retroTn transcripts are poorly polyadenylated [17]. The secondary structures of hps containing multiple inverted repeats are likely variable and complex with frayed ends (Fig. 5). Previous in vitro assays suggest that Dcr2 alone can bind and processively cleave blunt dsRNAs. However, Dcr2 requires a co-factor, Loqs-PD, to process dsRNAs with frayed termini presumably because Loqs-PD allows Dcr2 to bind a substrate internally [56]; Loqs-PD is cytoplasmic in *Drosophila* culture cells [57]. Taken together, these data suggest a model in which nuclear retained blunt-ended, fully complementary retroTn precursors can be processed in the nucleus by Dcr2 alone while more complicated hp precursors requiring Loqs-PD are cleaved in the cytoplasm by Dcr2 (Fig. 8). This model is supported by our observations that esiRNAs map the entire length of retroTns (Fig. 3d). Additionally, previous work shows that R2D2 and Dcr2 aggregate in cytoplasmic D2 bodies together with hps [27].

This model predicts that depletion of Loqs-PD would only affect cleavage of hps precursors and the levels of hp-derived esiRNAs, but not esiRNAs generated from retroTns. Zhou et al. previously reported that depletion of Loqs isoforms reduced the number of esiRNAs derived from both hps and Tns [58]; however, close examination of the data reveal that retroTn-mapping esiRNAs were unaffected by Loqs knockdown. The most notably affected Tn, Proto-P, is not regulated by the esiRNA pathway [59].

## Conclusions

Our data support a novel model in which esiRNAs are differentially processed from retroTn and hp precursors; retroTn precursors are processed by Dcr2 in the nucleus, while biogenesis of esiRNAs from hp precursors occurs in the cytoplasm. Additionally, Dcr2 clearly interacts with the CCC in the nucleus, but not in the cytoplasm. The CCC indirectly affects esiRNAs biogenesis by regulating Dcr2 substrate levels and directing cellular localization of retroTn and hp RNAs. These data contribute significantly to our understanding of Dcr2 dependent esiRNA production in *Drosophila* culture cells, but questions regarding Dcr2-CCC complex assembly and function remain. Future studies investigating the role of the Dcr2-CCC complex in both mRNA 3’ end processing and retroTn dsRNA processing will further elucidate molecular details of how these proteins function in *Drosophila* culture cells.

## Additional files

Additional file 1: Taqman assays and primers. Taqman assay ID #s and targets are shown in the top table. Targets of hp and Tn primers are shown in the middle table together with their sequences, position targeted within the Tn and references to their original use. Control GAPDH primers and primers used to assess sop RNA misprocessing are shown in the bottom table. (PDF 67 kb)

Additional file 2: Mass spectrometry (MS) identifies Symplekin binding partners. (A) Endogenous Symplekin was immunoprecipitated from crude nuclear extracts and bound proteins were visualized on an SDS-PAGE gel stained with coomassie blue (lane 3). Markers (Mar, lane 1) are labeled in KDa (left). α-Myc (lane 2) is a non-specific antibody control. Individual bands were cut from the gel and proteins identified by MS. The primary protein in each band is labeled. (B) MS data for each gel slice (samples a-i) is represented with gene name, Flybase ID and known functions of each identified protein. (PDF 3228 kb)

Additional file 3: Refined nuclear cytoplasmic/nuclear fractionation protocol. (A) S2 cells are first swelled in hypotonic buffer and then lysed with a tight-fitting dounce. Cell lysate is then centrifuged to separate the cytoplasm from the nuclei. The crude cytoplasmic fraction is purified by ultracentrifugation. The crude nuclear fraction is further purified by ultracentrifugation through a layered sucrose cushion. (B) This protocol results in excellent separation of S2 cytoplasm and nuclear material. Western blot of MEK 1/2 (cytoplasmic control) and H3 (nuclear controls) show no nuclear contamination in the cytoplasmic fraction and vice versa. (PDF 900 kb)

Additional file 4: Immunofluorescence shows Dcr2 in the nucleus. Immunofluorescence of *Drosophila* culture cells with anti-Dcr2 and anti-Symp antibodies shows both Dcr2 and Symplekin co-localizing with the DAPI stained nucleus. (PDF 130 kb)

Additional file 5: Work flow for high throughput sequencing and small RNA analysis (SMACR). (A) *Drosophila* cells were individually depleted of Dcr2, CPSF73, Symplekin, and GFP (or LacZ). An additional fifth sample was untreated. The untreated and GFP samples represent controls. RNA was isolated from each sample and fractionated into RNAs > than 200 nts and RNAs < 200 nts. Each sample was depleted of appropriate rRNAs followed by library construction in triplicate. RNA-seq was performed at Washington University while smRNA-seq was performed at University of Missouri-St. Louis. (B) Adapters were trimmed from the raw reads followed by filtering out all small RNAs larger than 30 nts. Small RNAs were mapped using Bowtie and were then sorted by feature: miRNA, transposon, hairpin, or non-coding RNA. The normalized read count of each unique small RNA mapping to each feature was calculated together with 3’ and 5’ and size abundance. (PDF 293 kb)

Additional file 6: HTS statistics. (A) Sample name, total number of reads, percent of reads mapping, read depth (# mapped reads/*Drosophila* transcriptome size (30.1 Mba)), percent unique and percent non-unique reads are shown for technical triplicates of each sample. A Student’s *T*-test was used to determine if the observed differences in percentages of non-uniquely mapping reads between samples was statistically significant. Corresponding *p* values are shown in the last column. (B) Total number of reads and percent or reads mapping when zero mismatches are allowed (left) and one mismatch is allowed (right) for three technical triplicates and one biological replicate (BR) of each sample. (PDF 54 kb)

Additional file 7: Physical characteristics of miRNAs, Tn- and hp-derived esiRNAs in Symplekin, CPSF73, Dcr2 knockdown and control samples. Mapped siRNAs were sorted by type and filtered by size (21-24 nts) (A), 3’ base (B), and 5’ base (C) for each sample. The abundance of normalized read counts in each category was then summed and the percentage of each individual category was calculated for all samples. Percentages for each category were then plotted. (PDF 208 kb)

Additional file 8: Esi1/2 precursors are polyadenylated. Polyadenylation status of CG44774 (Esi1) and CR18854 (Esi2) were assessed as described in [17]. These RNAs are more enriched in the Poly(A) + fraction than the polyadenylated Actin mRNA. Non-polyadenylated 18S rRNA is enriched in the poly(A)- fraction. (PDF 29 kb)

## Abbreviations

AS: Antisense
CCC: Core cleavage complex
CPSF: Cleavage and polyadenylation specificity factor
Dcr2: Dicer2
dsRNA: double stranded RNA
esiRNAs: endogenous small interfering RNAs
hp: hairpin
miRNAs: microRNAs
piRNAs: PIWI-interacting RNAs
retroTn: retrotransposons
S: Sense
SMACR: Sequence Mapping, Annotation, and Counting for smRNAs
Tn: Transposon
