# Supplementary material for: Drosophila melanogaster retrotransposon and inverted repeat-derived endogenous siRNAs are differentially processed in distinct cellular locations
Source: BMC Genomics. 2017 Apr 17;18:304. doi: 10.1186/s12864-017-3692-8 (PMC5392987; doi:10.1186/s12864-017-3692-8)
Supplement: Supplementary file 1 — Taqman assays and primers. Taqman assay ID #s and targets are shown in the top table. Targets of hp and Tn primers are shown in the middle table together with their sequences, position targeted within the Tn and references to their original use. Control GAPDH primers and primers used to assess sop RNA misprocessing are shown in the bottom table. (PDF 67 kb) [file 12864_2017_3692_MOESM1_ESM.pdf]

Additional file 1

Taqman Assays

Small RNA Target	Assay ID number
2S	001766
esi2.1	CSN1KEF
esi1.2	CSPACQN
Dm297	CSAAYTW
mdg1	CSBJWZ4
Mir2a	000261

Hairpin and Transposon Precursor Primers

Target Sequence	5' to 3' Sequence	Position (bp)	Reference
Dm297-RT Forward	ggcagacagagacggag	4629:4645	Russo et. al 2016
Dm297-RT Reverse	cgacttcttcttcttcaagc	4673:4693	Russo et. al 2016
Dm297-env Forward	gacaccactatacacaccac	6269:6289	Russo et. al 2016
Dm297-env Reverse	ctcaataatgtcgttgtg	6317:6335	Russo et. al 2016
Blood-ORFII Forward	cgtaaaaggcgaatcgcctg	2534:2554	Russo et. al 2016
Blood-ORFII Reverse	gctgcttacgcatactgtc	2624:2643	Russo et. al 2016
Blood-RT Forward	cctataccaacagatgccgac	4647:4668	Russo et. al 2016
Blood-RT Reverse	caaagcctcgttaagtggcg	4726:4746	Russo et. al 2016
Mdg-ORFII Forward	ctgagatcggtgaggatatcg	2053:2074	Russo et. al 2016
Mdg-ORFII Reverse	cgggtaatgttattaccgctg	2133:2154	Russo et. al 2016
Mdg-RT Forward	gtaaacaagcatgtggagcg	4824:4844	Russo et. al 2016
Mdg-RT Reverse	ctcctgctctgtagtggac	4923:4942	Russo et. al 2016
Jockey-gag Forward	acctatcctcaccccttctc	776:795	Russo et. al 2016
Jockey-gag Reverse	tgctccatattctccgtttcag	919:897	Russo et. al 2016
Jockey-RT Forward	gtggacattgataatgccacaag	2841:2864	Russo et. al 2016
Jockey-RT Reverse	ggaagttgaagtggtctgaag	2922:2943	Russo et. al 2016
Juan-ORFI Forward	ctgtgagttctacacgtacgatac	499:522	Russo et. al 2016
Juan-ORFI Reverse	cctaggttttgtagcatggatttg	586:609	Russo et. al 2016
Juan-RT Forward	gcgcaatgtaaaaacatatccg	2082:2104	Russo et. al 2016
Juan-RT Reverse	ctgtgagcagttgacaaccac	2168:2189	Russo et. al 2016
AY119029 (esi2.1) F	ccagggcgctacattcaata	multiple	Marques et. al 2010
AY119029 (esi2.1) R	caaacacccacacacatacaca	multiple	Marques et. al 2010
CG18854 (esi1.2) F	caaggctagggctcgtca	multiple	Marques et. al 2010
CG18854 (esi1.2) R	ggtgctgcgcataccttt	multiple	Marques et. al 2010

Additional Primers

Target Sequence	5' to 3' Sequence	Reference
GAPDH F	CGTTCATGCCACCACCGCTA	Russo et. al 2016
GAPDH R	CCACGTCCATCACGCCACAA	Russo et. al 2016
sop '3 UTR F	GGATTGCTACACCTCGGCCCG	Tatomer et. al 2014
sop '3 UTR R	CTACAACAGAATCTCCAAATCGACC	Tatomer et. al 2014

Additional file 8
