# Supplementary material for: Drosophila melanogaster retrotransposon and inverted repeat-derived endogenous siRNAs are differentially processed in distinct cellular locations
Source: BMC Genomics. 2017 Apr 17;18:304. doi: 10.1186/s12864-017-3692-8 (PMC5392987; doi:10.1186/s12864-017-3692-8)
Supplement: Supplementary file 2 — Mass spectrometry (MS) identifies Symplekin binding partners. (A) Endogenous Symplekin was immunoprecipitated from crude nuclear extracts and bound proteins were visualized on an SDS-PAGE gel stained with coomassie blue (lane 3). Markers (Mar, lane 1) are labeled in KDa (left). α-Myc (lane 2) is a non-specific antibody control. Individual bands were cut from the gel and proteins identified by MS. The primary protein in each band is labeled. (B) MS data for each gel slice (samples a-i) is represented with gene name, Flybase ID and known functions of each identified protein. (PDF 3228 kb) [file 12864_2017_3692_MOESM2_ESM.pdf]

A.

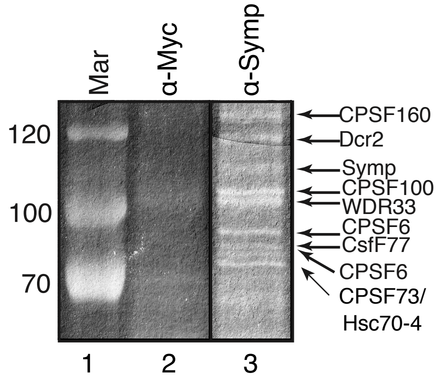

B.

Sample	Name	FlybaseID	MW (Da)	# of Peptides	Function
a	CPSF160	FBgn0024698	164.7	33	3' end processing
b	Dicer-2	FBgn0034246	197.8	23	siRNA
c	Symp	FBgn0037371	132.1	47	3' end processing
d	CPSF100	FBgn0027873	85.4	31	3' end processing
e	WDR33	FBgn0046222	90.5	29	3' end processing
f	CPSF6	FBgn0035872	71.1	29	3' end processing/ poly(A) site selection
g	CstF77	FBgn0003559	84.5	32	3' end processing
h	CPSF6	FBgn0035872	71.1	22	3' end processing/ poly(A) site selection
i	CPSF73	FBgn0261065	76.8	20	3' end processing
	Hsc70-4	FBgn0266599	71.1	11	RISC loading
