# Supplementary figures and images for: Drosophila melanogaster retrotransposon and inverted repeat-derived endogenous siRNAs are differentially processed in distinct cellular locations
Source: BMC Genomics. 2017 Apr 17;18:304. doi: 10.1186/s12864-017-3692-8 (PMC5392987; doi:10.1186/s12864-017-3692-8)

Additional file 3

A.

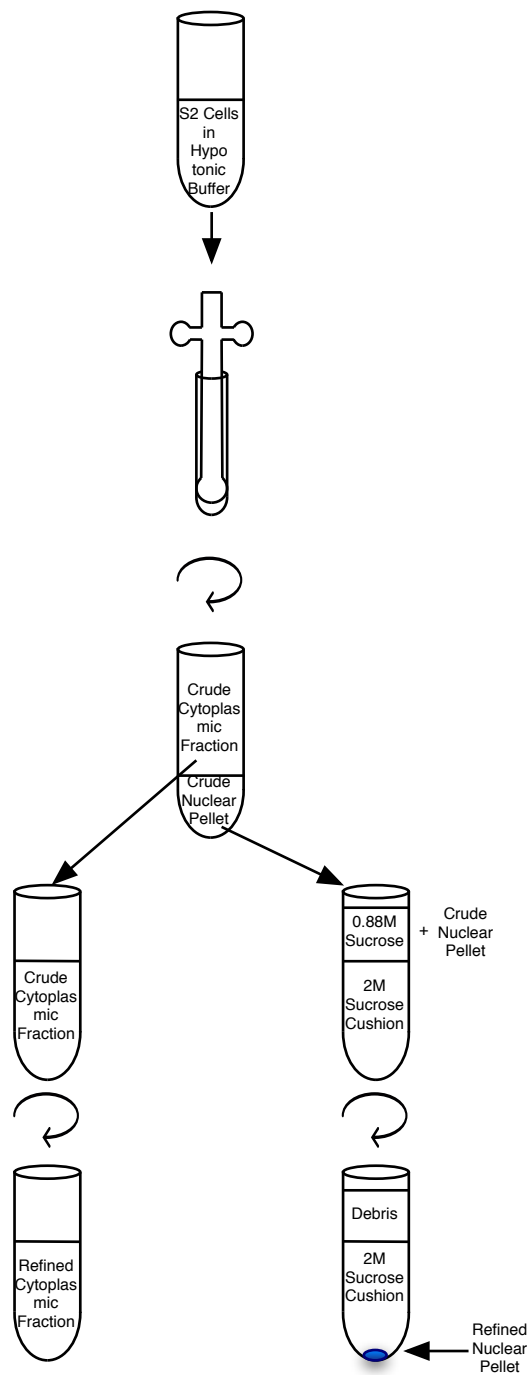

B.

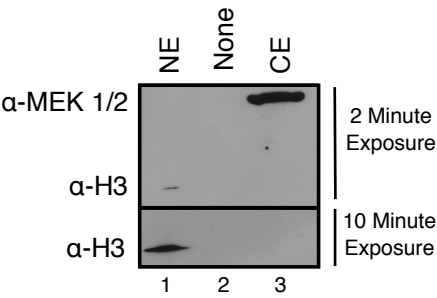

Supplement: Supplementary file 3 — Refined nuclear cytoplasmic/nuclear fractionation protocol. (A) S2 cells are first swelled in hypotonic buffer and then lysed with a tight-fitting dounce. Cell lysate is then centrifuged to separate the cytoplasm from the nuclei. The crude cytoplasmic fraction is purified by ultracentrifugation. The crude nuclear fraction is further purified by ultracentrifugation through a layered sucrose cushion. (B) This protocol results in excellent separation of S2 cytoplasm and nuclear material. Western blot of MEK 1/2 (cytoplasmic control) and H3 (nuclear controls) show no nuclear contamination in the cytoplasmic fraction and vice versa. (PDF 900 kb) [file 12864_2017_3692_MOESM3_ESM.pdf]

## Additional file 4

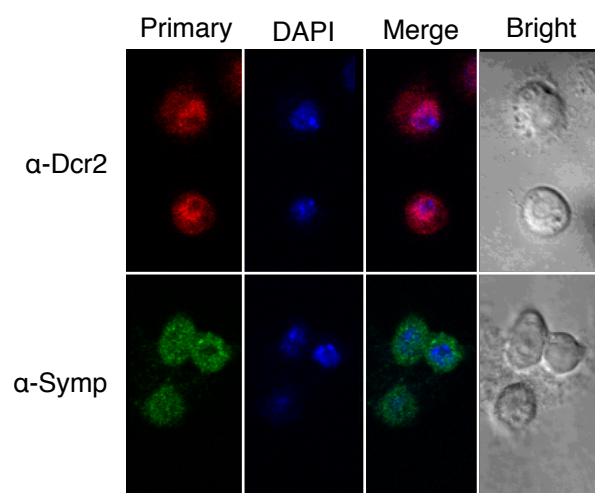

Supplement: Supplementary file 4 — Immunofluorescence shows Dcr2 in the nucleus. Immunofluorescence of Drosophila culture cells with anti-Dcr2 and anti-Symp antibodies shows both Dcr2 and Symplekin co-localizing with the DAPI stained nucleus. (PDF 130 kb) [file 12864_2017_3692_MOESM4_ESM.pdf]

## Additional file 5

A.

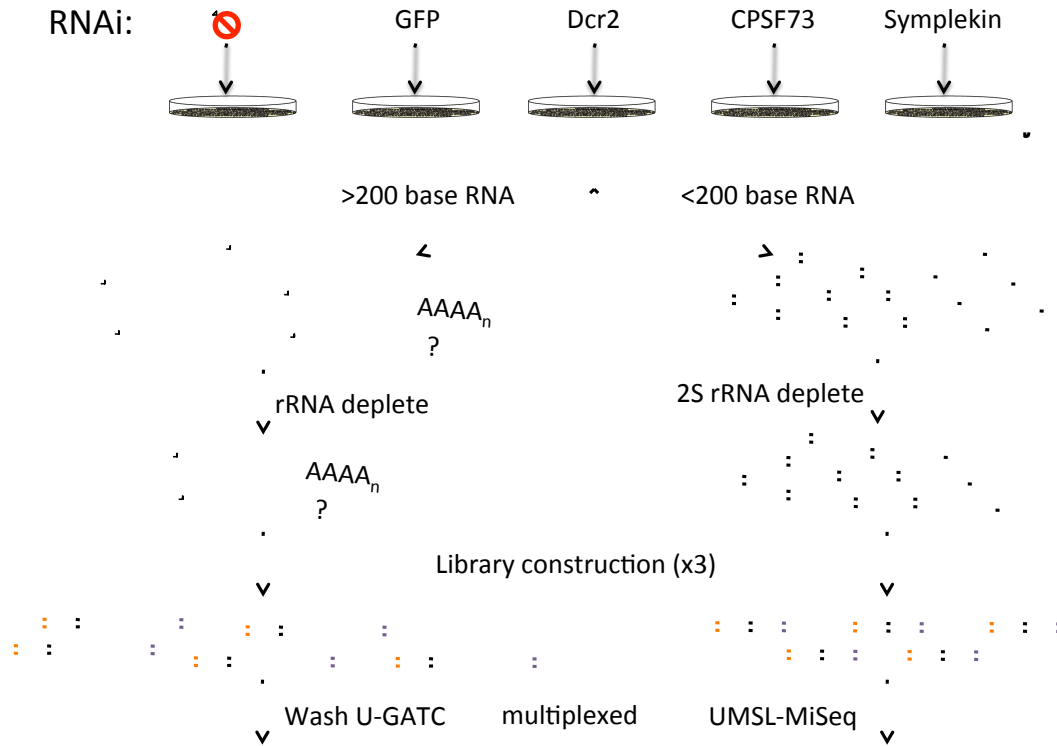

B.

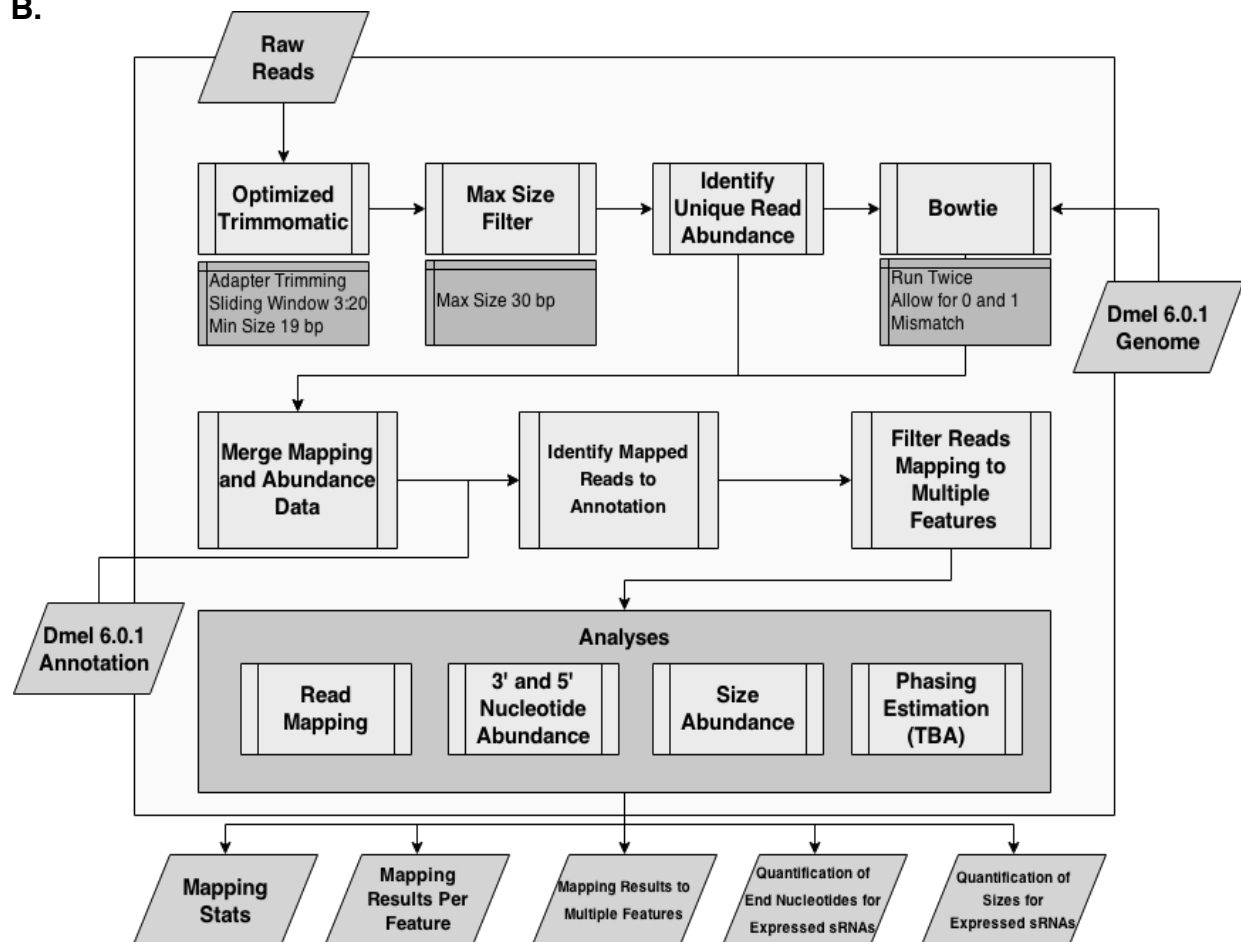

Supplement: Supplementary file 5 — Work flow for high throughput sequencing and small RNA analysis (SMACR). (A) Drosophila cells were individually depleted of Dcr2, CPSF73, Symplekin, and GFP (or LacZ). An additional fifth sample was untreated. The untreated and GFP samples represent controls. RNA was isolated from each sample and fractionated into RNAs > than 200 nts and RNAs < 200 nts. Each sample was depleted of appropriate rRNAs followed by library construction in triplicate. RNA-seq was performed at Washington University while smRNA-seq was performed at University of Missouri-St. Louis. (B) Adapters were trimmed from the raw reads followed by filtering out all small RNAs larger than 30 nts. Small RNAs were mapped using Bowtie and were then sorted by feature: miRNA, transposon, hairpin, or non-coding RNA. The normalized read count of each unique small RNA mapping to each feature was calculated together with 3’ and 5’ and size abundance. (PDF 293 kb) [file 12864_2017_3692_MOESM5_ESM.pdf]

Additional file 7

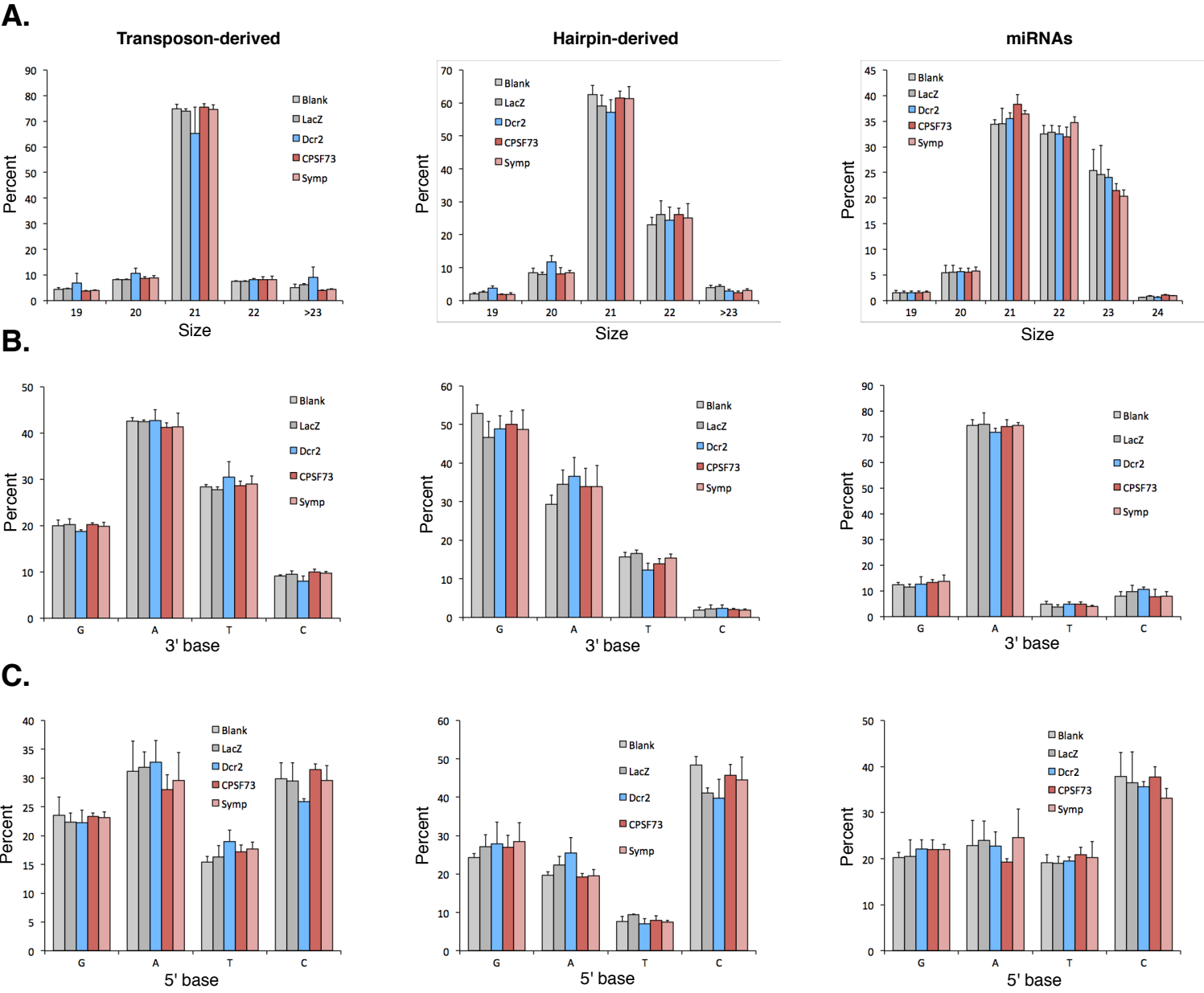

Supplement: Supplementary file 7 — Physical characteristics of miRNAs, Tn- and hp-derived esiRNAs in Symplekin, CPSF73, Dcr2 knockdown and control samples. Mapped siRNAs were sorted by type and filtered by size (21-24 nts) (A), 3’ base (B), and 5’ base (C) for each sample. The abundance of normalized read counts in each category was then summed and the percentage of each individual category was calculated for all samples. Percentages for each category were then plotted. (PDF 208 kb) [file 12864_2017_3692_MOESM7_ESM.pdf]

# Additional file 8

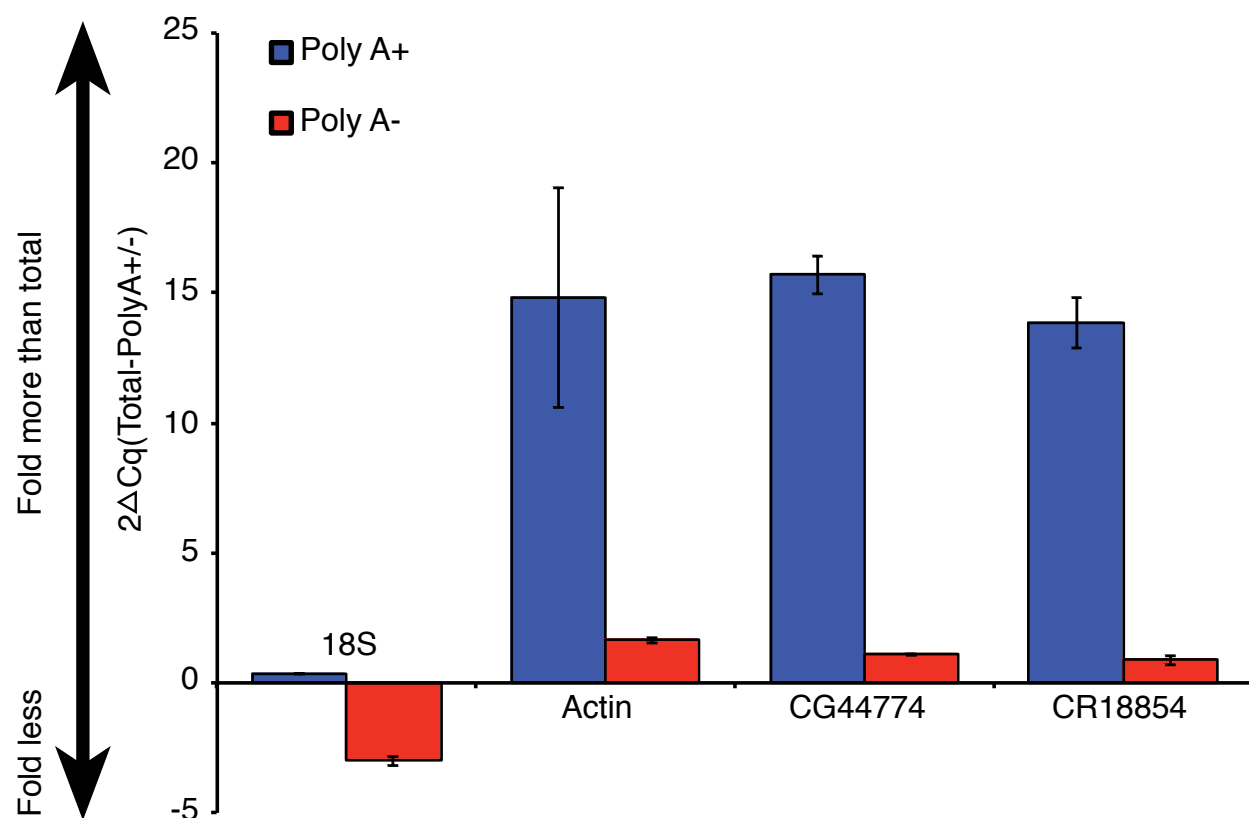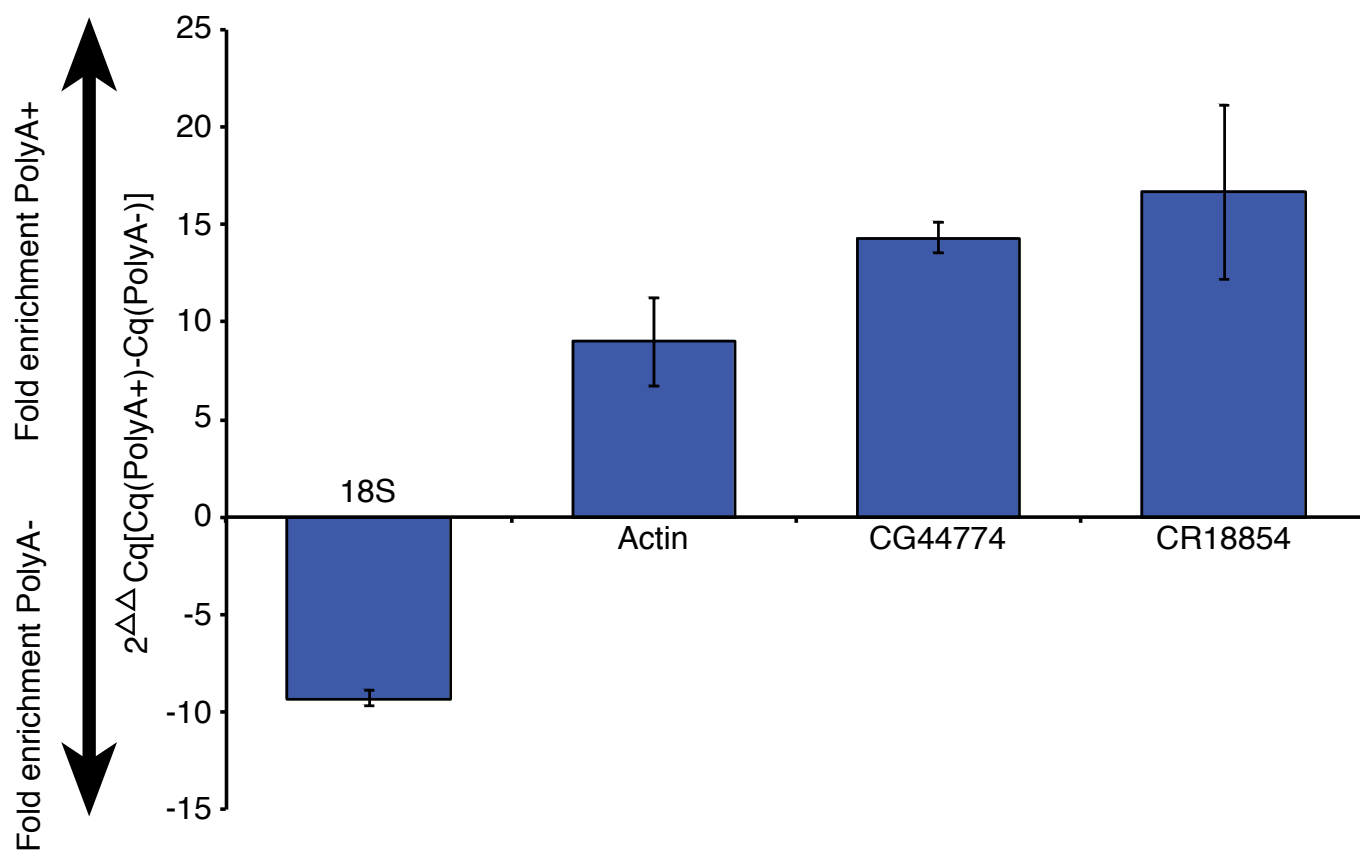

Supplement: Supplementary file 8 — Esi1/2 precursors are polyadenylated. Polyadenylation status of CG44774 (Esi1) and CR18854 (Esi2) were assessed as described in [17]. These RNAs are more enriched in the Poly(A) + fraction than the polyadenylated Actin mRNA. Non-polyadenylated 18S rRNA is enriched in the poly(A)- fraction. (PDF 29 kb) [file 12864_2017_3692_MOESM8_ESM.pdf]
