# Supplementary material for: Drosophila melanogaster retrotransposon and inverted repeat-derived endogenous siRNAs are differentially processed in distinct cellular locations
Source: BMC Genomics. 2017 Apr 17;18:304. doi: 10.1186/s12864-017-3692-8 (PMC5392987; doi:10.1186/s12864-017-3692-8)
Supplement: Supplementary file 6 — HTS statistics. (A) Sample name, total number of reads, percent of reads mapping, read depth (# mapped reads/Drosophila transcriptome size (30.1 Mba)), percent unique and percent non-unique reads are shown for technical triplicates of each sample. A Student’s T-test was used to determine if the observed differences in percentages of non-uniquely mapping reads between samples was statistically significant. Corresponding p values are shown in the last column. (B) Total number of reads and percent or reads mapping when zero mismatches are allowed (left) and one mismatch is allowed (right) for three technical triplicates and one biological replicate (BR) of each sample. (PDF 54 kb) [file 12864_2017_3692_MOESM6_ESM.pdf]

Additional file 6

A. RNA-seq

Sample	Total Reads	% Mapping	Read Depth	% Unique	% Non-Uniq	p-values
Blank1	24698945	98.8	82.1	58.3	41.7	0.112
Blank2	26712148	98.3	88.7	57.1	42.9	
Blank3	26318291	98.2	87.4	57.2	42.8	
LacZ1	30020327	98.5	99.7	57.0	43.0	
LacZ2	35561677	98.4	118.1	56.3	43.7	
LacZ3	26072570	98.4	86.6	56.4	43.6	
Dcr2-1	22668363	98.2	75.3	51.8	48.2	6.949E-05
Dcr2-2	23826043	98.0	79.2	51.3	48.7	
Dcr2-3	24236601	98.0	80.5	51.1	48.9	
CPSF73-1	27808742	98	92.4	58.2	41.8	0.521
CPSF73-2	22354433	98.1	74.3	56.6	43.4	
CPSF73-3	24986528	98.3	83.0	56.3	43.7	
Symp1	28837214	98.5	95.8	53.3	46.7	0.007
Symp2	25326064	98.2	84.1	53.2	46.8	
Symp3	31041518	97.2	103.1	51.8	48.2	

B. SiRNA-seq

Mismatch 0:

Sample	Total Reads	% Mapping
Blank1	296806	67.8
Blank2	257223	69.5
Blank3	144083	73.0
Blank4-BR	935588	84.8
CPSF73-1	328575	74.0
CPSF73-2	272747	78.2
CPSF73-3	292295	79.1
CPSF73-4-BR	857530	70.5
Dcr2-1	227654	71.8
Dcr2-2	312873	73.3
Dcr2-3	237995	71.7
Dcr2-4-BR	784740	81.7
LacZ1	390118	71.4
LacZ2	330015	73.1
LacZ3	329624	74.1
GFP-BR	928750	73.6
Sym1	468391	73.2
Sym2	332289	73.9
Sym3	389207	73.4
Sym4-BR	963513	79.0

Mismatch 1:

Sample	Total Reads	% Mapping
Blank1	296806	93.7
Blank2	257223	93.9
Blank3	144083	94.3
Blank4-BR	935588	96.9
CPSF73-1	328575	95.3
CPSF73-2	272747	95.3
CPSF73-3	292295	95.4
CPSF73-4-BR	857530	89.3
Dcr2-1	227654	95.5
Dcr2-2	312873	95.7
Dcr2-3	237995	95.6
Dcr2-4-BR	784740	95.1
LacZ1	390118	94.0
LacZ2	330015	94.0
LacZ3	329624	94.4
GFP-BR	928750	93.7
Sym1	468391	94.6
Sym2	332289	94.6
Sym3	389207	94.7
Sym4-BR	963513	94.3
